# Editorial: Phagocytes in Immunity: Linking Material Internalization to Immune Responses and Therapeutic Strategies

**DOI:** 10.3389/fimmu.2021.772256

**Published:** 2021-09-24

**Authors:** Johnathan Canton, Gregory D. Fairn, Valentin Jaumouillé

**Affiliations:** ^1^ Comparative Biology and Experimental Medicine, University of Calgary, Calgary, AB, Canada; ^2^ Department of Biology, Dalhousie University, Halifax, NS, Canada; ^3^ Molecular Biology and Biochemistry, Simon Fraser University, Burnaby, BC, Canada

**Keywords:** macrophage, dendritic cell, neutrophil, phagocytosis, efferocytosis, macropinocytosis, immunotherapy, host-pathogen interactions

In this collection, a series of primary research articles and reviews cover the impressive functional diversity of phagocytes in the maintenance of organismal homeostasis and immunity. Phagocytes, including neutrophils, macrophages and dendritic cells, are endowed with the unique capacity to internalize large quantities of exogenous material. This can occur through the receptor-dependent uptake of particulate material by phagocytosis or through the uptake of fluid-phase material by macropinocytosis. In both cases, the internalized material is processed and information is extracted allowing phagocytes to discern homeostatic debris from any potential threats. A failure in phagocyte function results in a departure from homeostasis. The study of phagocytes serves as a venue for interrogating fundamental questions in the biology of molecules, cells and organisms. Here we highlight the contributions to this Research Topic:

Both phagocytosis and macropinocytosis are initiated by receptors. One commonly studied phagocytic receptor is the Fcγ receptor (FcγR), which binds to antibodies attached to phagocytic targets. A prerequisite for FcγR signaling involves their clustering in the plane of the plasma membrane. Clustering is achieved when large, multivalent targets, such as immune complexes, come into contact with the phagocyte. In this collection, Bailey et al. demonstrate that, below a critical threshold, FcγR clustering can in fact be inhibitory, adding another layer to our understanding of the regulation of phagocytic receptor function. In many instances, including in the case of FcγR-mediated phagocytosis, transmembrane proteins called integrins are intimately involved, and indeed required, for efficient phagocytosis. Sun et al. describe the activation of and mechanisms by which integrins contribute to phagocytosis and phagocyte function.

Phagocytes encounter a diverse array of targets. Indeed, targets may be completely immobile, or, as is the case for many pathogens, may be swimming away at high speed! Targets may be less than a micron across, or may be filamentous, extending many microns away from the phagocyte. Yet, phagocytes have evolved mechanisms for dealing with the vast diversity of targets they could encounter. Baranov et al. discuss in detail how particle size and shape affect phagocytosis.

One commonly encountered phagocytic target is homeostatic debris. Endowed with an array of receptors, phagocytes can bind to and internalize dead and damaged cells. Internalization is followed by digestion of the homeostatic debris into exportable components, such as amino acids, nucleotides and lipids which are transferred out of the phagosome by transmembrane transporters for recycling or export from the cell. This process is best exemplified by the clearance of dead and dying cells through a process known as efferocytosis. Yin and Heit provide a comprehensive review of efferocytosis and discuss its salient features including phagocyte metabolism during efferocytosis, antigen extraction from internalized cells and the coupling of efferocytosis to the resolution of inflammation. The clearance of spent cells is not restricted to professional phagocytes. Tissue-resident non-myeloid cells can harbor phagocyte receptors and play key roles in the maintenance of tissue homeostasis. Kwon and Freeman describe how retinal pigment epithelial (RPE) cells contribute to the optimal functioning of tissues in the eye. Finally, the scavenging activity of phagocytes is tunable. They remain responsive to external cues and can therefore regulate both phagocytic and macropinocytic activity in a context-dependent manner. Decker et al. describe how specialized pro-resolving mediators contribute to the resolution of inflammation by modulating the phagocytic and efferocytic activity of phagocytes.

Phagocytes can also contribute to immunity through the direct clearance of pathogens by phagocytosis. Interestingly, Kornstädt et al. show that during inflammation, mast cells serve as critical regulators of the phagocytic activity of neighboring phagocytes. Despite phagocyte specialization for rapid and contained killing, many pathogens have evolved means of either evading or indeed parasitizing phagocytes. Gioseffi et al. review how pathogens parasitize phagocyte membrane trafficking pathways to optimize their own survival.

Phagocytosis and macropinocytosis generate nascent vacuoles, which must mature into degradative organelles akin to endosomal maturation. The harsh environment within these compartments allows for the killing of microbial threats, the extraction of antigen for presentation to cells of the adaptive immune system, and the delivery of pathogen-associated molecular patterns (PAMPs) to intracellular pattern recognition receptors (PRRs). Critical for this maturation is the fusion of phagosomes/macropinosomes with lysosomes, which deliver hydrolases and promote lumen acidification. Nguyen and Yates discuss the current understanding of the molecular mechanisms of the fusion of phagosomes with lysosomes. Protein S-acylation is a post-translational modification that alters the function of many cellular proteins including those involved in phagocytosis and phagosome maturation. Dixon et al., discuss what is known and highlight many questions that remain to understand this modification during these processes. Additionally, Kawai et al. describe endocytic structures marked by Rab10 that occur in response to macropinocytic stimuli but are instead tubular in nature.

Altering and exploiting the adaptive immune response to combat tumors has been a popular area for many years. Chen et al. and Britt et al. discuss how manipulating the innate immune response and in particular harnessing macrophages and phagocytosis can be beneficial as a targeted cancer therapy. The direct clearance of malignant tumor cells by macrophages and presentation of cell-associated antigen by dendritic cells to T cells have both emerged as areas of intense study in cancer research. As we continue to learn about phagocytes, new avenues for understanding the basic biology of organisms and leveraging that information for future therapies will undoubtedly continue to emerge.

## Author Contributions

All authors listed have made a substantial, direct, and intellectual contribution to the work and approved it for publication.

## Conflict of Interest

The authors declare that the research was conducted in the absence of any commercial or financial relationships that could be construed as a potential conflict of interest.

## Publisher’s Note

All claims expressed in this article are solely those of the authors and do not necessarily represent those of their affiliated organizations, or those of the publisher, the editors and the reviewers. Any product that may be evaluated in this article, or claim that may be made by its manufacturer, is not guaranteed or endorsed by the publisher.

